# A Covering Method for Detecting Genetic Associations between Rare Variants and Common Phenotypes

**DOI:** 10.1371/journal.pcbi.1000954

**Published:** 2010-10-14

**Authors:** Gaurav Bhatia, Vikas Bansal, Olivier Harismendy, Nicholas J. Schork, Eric J. Topol, Kelly Frazer, Vineet Bafna

**Affiliations:** 1 Department of Computer Science and Engineering, University of California San Diego, La Jolla, California, United States of America; 2 Harvard-MIT Division of Health Sciences and Technology, Cambridge, Massachusetts, United States of America; 3 Scripps Translational Science Institute, La Jolla, California, United States of America; 4 Department of Pediatrics, University of California San Diego, La Jolla, California, United States of America; 5 Institute for Genomic Medicine, University of California San Diego, La Jolla, California, United States of America; University of Zurich, Switzerland

## Abstract

Genome wide association (GWA) studies, which test for association between common genetic markers and a disease phenotype, have shown varying degrees of success. While many factors could potentially confound GWA studies, we focus on the possibility that multiple, rare variants (RVs) may act in concert to influence disease etiology. Here, we describe an algorithm for RV analysis, RareCover. The algorithm combines a disparate collection of RVs with low effect and modest penetrance. Further, it does not require the rare variants be adjacent in location. Extensive simulations over a range of assumed penetrance and population attributable risk (PAR) values illustrate the power of our approach over other published methods, including the collapsing and weighted-collapsing strategies. To showcase the method, we apply RareCover to re-sequencing data from a cohort of 289 individuals at the extremes of Body Mass Index distribution (NCT00263042). Individual samples were re-sequenced at two genes, FAAH and MGLL, known to be involved in endocannabinoid metabolism (187Kbp for 148 obese and 150 controls). The RareCover analysis identifies exactly one significantly associated region in each gene, each about 5 Kbp in the upstream regulatory regions. The data suggests that the RVs help disrupt the expression of the two genes, leading to lowered metabolism of the corresponding cannabinoids. Overall, our results point to the power of including RVs in measuring genetic associations.

## Author Summary

We focus on the problem of detecting multiple rare variants (RVs) that act together to influence disease phenotypes. In considering this problem, we argue that the detection of causal rare variants must necessarily be different from typical single-marker analysis used for common variants and propose a novel algorithm, RareCover, to accomplish this analysis. RareCover combines a disparate collection of RVs, each with very low effect and modest penetrance. Extensive simulations over a range of values for penetrance and population attributable risk (PAR) illustrate the power of our approach over other published methods, including the collapsing and weighted-sum strategies. To showcase the method, we applied RareCover to data from 289 individuals at the extremes of Body Mass Index distribution (NCT00263042), sequenced around the FAAH and MGLL genes. RareCover analysis identified exactly one significantly associated region in each gene, each about 5Kbp in the upstream regulatory regions. The data suggests that the RVs help disrupt the expression of the two genes leading to lowered metabolism of the corresponding endocannabinoids previously linked with obesity. Overall, our results point to the power of including RVs in measuring genetic associations, and suggest that whole genome, DNA sequencing-based association studies investigating RV effects are feasible.

## Introduction

The Common Disease, Common Variant (CDCV) hypothesis [1]–[3] postulates that the etiology of common diseases is mediated by commonly occurring genomic variants in a population. This has served as the basis for genome wide association (GWA) studies that test for association between individual genomic markers and the disease phenotype. Using genome-wide panels of common SNPs, GWA studies have been successful in identifying hundreds of statistically significant associations for many common diseases as well as several quantitative traits [4]–[7]. Nevertheless, the success of GWA studies has been mixed. Significant genetic loci have not been detected for several common diseases that are known to have a strong genetic component [4]. Additionally, for many common diseases, associations discovered in GWA studies can account for only a small fraction of the heritability of the disease. While many factors could potentially confound GWA studies, we focus on the possibility that multiple, rare variants may act in concert to influence disease etiology.

The alternative to the CDCV hypothesis, the ‘Common Disease, Rare Variant (CDRV)’ hypothesis has been the topic of much recent debate [8], and has shown promise in explaining disease etiology in multiple studies. For example, rare variants (RVs) have been implicated in reduced sterol absorption and, consequently, lower plasma levels of LDL [9], [10] and colorectal cancer [11]. While some studies have shown RVs to increase risk, a recent study indicates that RVs also act ‘protectively’, with multiple RVs in renal salt handling genes showing association with reduced renal salt resorption and reduced risk of hypertension [12]. Additionally, rare mutations in IFIH1 have been shown to act protectively against type 1 diabetes [13].

The aforementioned studies and others focused on re-sequencing of the coding regions of candidate genes using Sanger sequencing (see Table 1 in Schork et al. [8] for a summary). Recent technological advances in DNA sequencing have made it possible to re-sequence large stretches of a genome in a cost-effective manner. This is enabling large-scale studies of the impact of RVs on complex diseases. However, several properties of rare variants make their genetic effects difficult to detect with current approaches. Bodmer and Bonilla provide an excellent review of the properties of RVs, and the differences between rare, and common variant analysis [14]. As an example, if a causal variant is rare (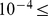 MAF 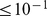), and the disease is common, then the allele's Population-Attributable-Risk (PAR), and consequently the odds-ratio (OR), will be low. Additionally, even highly penetrant RVs are unlikely to be in Linkage Disequilibrium (LD) with more common genetic variations that might be genotyped for an association study of a common disease. Therefore, single-marker tests of association, which exploit LD-based associations, are likely to have low power. If the CDRV hypothesis holds, a combination of multiple RVs must contribute to population risk. In this case, there is a challenge of detecting multi-allelic association between a locus and the disease.

Methods to detect such associations are only just being developed. A natural approach is a collapsing strategy, where multiple RVs at a locus are collapsed into a single variant. Such strategies have low power when ‘causal’ and neutral RVs are combined (See for example, Li and Leal [15]). Madsen and Browning have recently proposed a weighted-sum statistic to detect loci in which disease individuals are enriched for rare variants [16]. In their approach, variants are weighted according to their frequency in the unaffected sample, with low frequency variants being weighted more heavily. Each individual is scored as a sum of the weights of the mutations carried. The test then determines if the diseased individuals are weighted more heavily than expected in a null-model. Madsen and Browning show that with 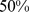 of variants in a group being causal and a combined odds ratio 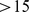, the weighted-sum statistic detects associations with high power. While effective, this approach depends upon the inclusion of high proportion of causal rare variants in the formation of the test statistics and strong penetrance to detect significant association. In their simulations, the PAR of the locus is partitioned equally among all variants, an assumption that may not always hold.

The Combined Multivariate and Collapsing Method (CMC), proposed by Li and Leal, combines variants into groups based upon predefined criteria (e.g. allele frequency, function) [15]. An individual has a ‘1’ for a group if any variant in the group is carried and a ‘0’ otherwise. The CMC approach then considers each of the groups in a multivariate analysis to explain disease risk. This combination of the collapsing approach and multivariate analysis results in an increase of power over single-marker and multiple marker approaches. However, as Li and Leal point out, the method relies on correct grouping of variants. The power is reduced as functional variants are excluded and non-functional variants are included in a group. Assignment of SNPs to incorrect groups may, in fact, decrease power below that attainable through single marker analysis. Indeed, a recent analysis by Manolio and colleagues suggests that new methods might be needed when the causal variants have both low PAR and low penetrance values [17].

Here, we focus on a model-free method, RareCover, that collapses only a subset of the variants at a locus. Informally, consider a locus 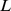 encoding a set 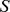 of rare variants. RareCover associates 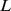 with a phenotype by measuring the strongest possible association formed by collapsing any subset 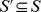 of variants at 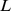. At first glance, such an approach has many problems. First, selecting an optimal subset of SNPs is computationally intensive, scaling as 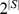. We show that a greedy approach to selecting the optimal subset scales linearly, making it feasible to conduct associations on a large set of candidate loci.

A second confounding factor is that the large number of different tests at a locus increase the likelihood of false association. The adjustment required to control the type I error could decrease the power of the method. However, extensive simulations show otherwise. Our results suggest that moderately penetrant alleles 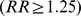 with small PAR 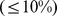, and moderately sized cohorts (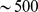 cases and 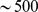 controls) are sufficient for RareCover to detect significant association. This compares well with the current power of single-marker GWA studies on common variants, and outperforms other methods for RV detection.

We also applied RareCover to the analysis of two genes, FAAH, and MGLL, in the endocannabinoid pathway in a large sequencing study of obese and non-obese individuals. The endocannabinoid pathway is an important mediator of a variety of neurological functions [18], [19]. Endocannabinoids, acting upon CB1 receptors in the brain, the gastrointestinal tract, and a variety of other tissues, have been shown to influence food intake and weight gain in animal models of obesity. Using a selective endocannabinoid receptor (CB1) antagonist, SR141716 (Rimonanabt; Sanofi-Synthelabo) leads to reduced food intake in mice. Correspondingly, elevation of leptin levels have been shown to decrease concentrations of endogenous CB1 agonists, Anandamide, and 2-AG in mice, thereby reducing food-intake [20]. The FAAH and MGLL enzymes serve as regulators of endocannabinoid signaling in the brain [21], by catalyzing the hydrolysis of endocannabinoid including anandamide (AEA), and 2-AG. Gene expression studies in lean and obese women show significantly decreased levels of AEA and 2-AG, as well as over-expression of CB1 and FAAH in lean, as opposed to obese women [22]. While evidence points to a genetic association of these loci with obesity, multiple recent studies using common SNPs in the FAAH region have failed to confirm an association [23]–[26]. A Pro129Thr polymorphism was tentatively associated with obesity in a cohort of Europe and and Asian ancestry, but has not been replicated in other data [27].

We tested the hypothesis that multiple, rare alleles at these loci are associated with obesity. We have used unpublished (submitted) data from Frazer and colleagues, where the FAAH (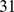
Kbp) and MGLL (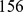
Kbp) regions were re-sequenced using next generation technologies in 148 obese and 150 non-obese individuals taken as extremes of the body mass index distribution from subjects in a large clinical trial (the CRESCENDO cohort, NCT00263042). The resequencing identified a number of common, and rare variants in the region. We applied RareCover to determine if multiple RVs, i.e., allelic heterogeneity, mediated the genetic effects of FAAH and MGLL on obesity. RareCover identified a single region at each locus with permutation adjusted p-values of 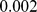 and 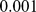. In each case, the significant locus was immediately upstream of the gene, consistent with a regulatory function for the rare variants.

## Methods

### Modeling RV association

We define a locus as a genomic region of fixed size (nucleotides). Let 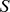 denote the set of RVs in the locus. We abuse notation slightly by using 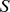 to also denote the locus itself. A case-control study at 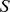 includes a set of individual genotypes. For genotype 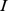, and RV 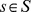, let 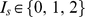 denote the number of minor alleles that genotype 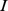 carries for variant 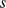. Extending the notation to subsets, 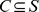 of RVs, define 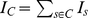. For a subset 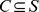, denote a *union-variant* 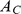 as follows: individual 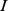 has the allele 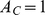 if and only if 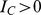. Otherwise, 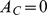. The union-variant is a virtual construct that helps combine the effect of multiple RVs. Let 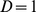 (respectively, 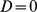) represent the case (respectively, control) status of an individual.

For an individual chosen at random, and 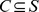, let Xcorr 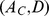 denote an association test statistic between the union-variant 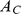 and the disease status 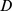. Here, we will use Pearson's 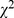 as the test-statistic, but the method remains unchanged for other measures. Using this notation, the collapsing strategy described by Li and Leal [15] uses the test-statistic Xcorr 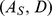 to associate a locus 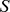 with the disease. Instead, we define the association statistic for locus 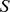 by
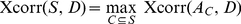
(1)


### The RareCover method

Our method, RareCover, accepts a locus containing a set 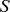 of RVs in a window of fixed size (nucleotides). It returns the test-statistic, Xcorr 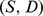, a 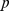-value on the statistic, and the subset 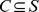 of RVs that contribute to the union variant. The window size 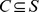 is a parameter. When the input locus is larger than the window size, RareCover looks at overlapping windows of size 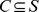, where each window is shifted one RV away from the previous window. For each window, the Xcorr statistic is output, along with a non-adjusted 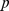-value.

The computation for the Xcorr statistic on a single window is described in the Algorithm below. Given a set 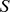 of RVs over 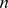 individuals, the naive computation for computing Xcorr 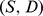 needs 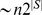 computations. A reduction from the MAX-COVER problem can be used to show that the problem is NP-hard, indicating that no provably efficient algorithm is likely [28]. Similar reductions can also be used to show the hardness result for a variety of other proposed association statistics. Therefore, we employ a greedy heuristic that is fast (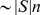 computations), and does well in practice. In each step, (see Algorithm), we select the variant that adds the most to the statistic, until no further improvement is possible. On a standard Linux workstation, the computation is fast, about 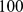 windows per second.

**procedure** RareCover (*S*, *Q*)

 Set 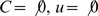

 repeat

  Set 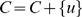

  Select 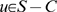 that maximizes Xcorr 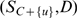

 while 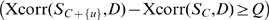

 return Xcorr 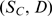.

*The* RareCover *method for detecting locus association*. 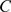 describes the current subset of ‘causal’ SNPs. Initially 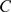 is empty. In each iteration, the RV 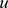 that maximizes the test statistic is chosen, and added to 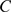. When the improved statistic 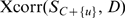 is not significantly better than the current statistic (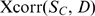), the method stops, and outputs 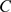.

#### Computing significance

While the test-statistic is a 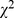 test on the union-variant 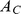, significance cannot be computed directly, as 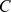 is optimized over many possibilities. The multiple-tests will increase the statistic for non-associated loci as well. We compute significance by applying RareCover to permutations of the case-control genotypes.

**Figure 1:**
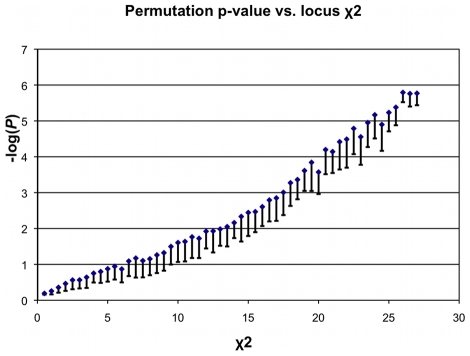
Permutation 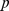-values versus the 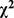 statistic value on the union-variant 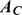. The mean of the empirical 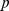-values (obtained by permuting cases and controls) were plotted against each value of the 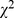 statistic obtained over many tests over the entire range of simulation parameters, by varying sample size 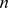, locus PAR, and penetrance. As 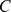 is the most significant subset among many possible subsets, the theoretical 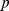-value suggested by the 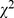 distribution cannot be used directly. However, the plot shows that the locus 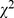 value correlates tightly with the 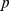-value, implying that the union 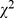 statistic can be used to filter the significant windows with no loss of power. The saturation at the ends is due to the number of trial being limited to 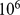.

The number of permuted trials required to achieve genome-wide significance can be large. We make the computation tractable using two ideas: first, empirical tests show that the 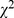 statistic on 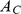 correlates tightly with the permutation 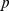-value (Figure 1). Note that the saturation at the end is due to limited trials. Let 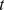 denote the value of Xcorr for a window, 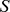. When 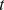 is less than a pre-determined threshold (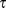), no permutation test is done, as the window is unlikely to be significant. When 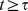, the statistic is recomputed after permuting case and control labels (default 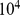 permutations), and a 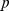-value is computed as the fraction of the permuted samples whose Xcorr value matches or exceeds 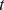. To save time on this computation, we run permutations in a data-driven fashion. We run a maximum of 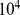 permutations, but stop as soon as we obtain 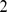 samples for which the RareCover statistic exceeds 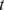.
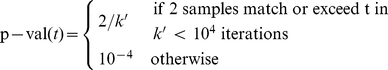
(2)Both, the parameter 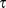, and the maximum number of iterations can be adjusted, based on desired level of significance, and the size of the genomic region. Here, we set parameter 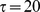, which corresponds to a permutation adjusted 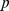-value of 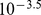 in Figure 1. This ensures fast computation with no loss of power (See Results).

#### RareCover for genic regions

RareCover can also be applied to a locus containing a single gene. The definition of a gene varies; it sometimes includes only the coding exons, or it can include all exons including UTRs, and even regulatory regions. While the scanning window approach of RareCover can be applied unchanged for any genic locus, we must correct for multiple windows at a locus. Given a genic locus, we permute cases and controls multiple times and score every window in the locus. Then, the adjusted (locus) 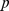-value of a window with Xcorr value 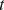 is the fraction of all permuted windows in the locus with an Xcorr score of 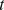 or higher.

#### RareCover parameters

RareCover is a model-free approach, and has only 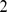 parameters: the window size 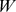, and a convergence cut-off 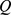 (see procedure above). Empirical tests by simulation show that the performance is similar despite large variations in window size 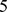-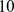
Kbp, as well as a choice of 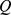. Hence, no explicit training was performed, and the parameters were set to 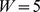
Kbp, and 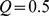. The performance of RareCover was extensively tested over a wide range of simulation parameters.

### Parameters for RV simulation

Consider a locus with a set 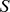 of rare-variants. Let a subset 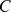 of RVs be *causal*, in the sense that a mutation at any 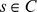 increases the likelihood of disease. For an individual, 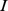, we use 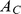 and 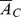 as short-forms of the events 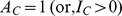, and 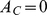, respectively. Similarly, events 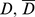 reflect case-control status for the individual. We work with the following 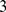 parameters for power calculations:

1. *Disease prevalence* in the population, denoted by 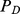.
2. *Penetrance* of the locus, denoted by 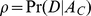

3. *Locus-PAR*, denoted by 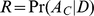

Note that the PAR for a variant is often described by the following (Ex:Bodmer and Bonilla, 1999 [14])
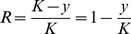
(3)where 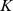 is the number of individuals with the phenotype, and 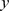 is the number of individuals that show the phenotype, but do not have the variant allele. In our terminology
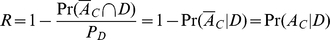



The choice of these parameters is intuitive as we expect an RV to have moderate penetrance, but very low PAR 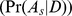. However, the multiple RVs in 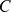 have roughly additive effect, leading to moderate locus-PARs. These parameters are tightly related to other, more common measures of locus association, such as the Odds-Ratio (OR), as shown below:
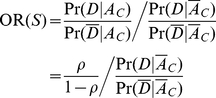
To compute 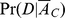, we start with a Bayesian relation for computing the likelihood of a genotype containing a causal RV as
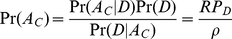
(4)Then,
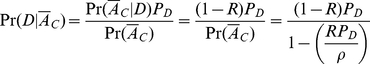
(5)and,
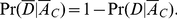
(6)


### Simulating constant sized populations (CP)

We simulate multiple case-control studies over a range 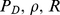. A simulation of 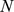 individuals begins with the division of the individuals into 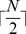 cases and 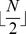 controls. Once this is done two additional steps take place.

1. Generate a set of RVs for the simulated locus containing causal, and neutral RVs.
2. Simulate the genotypes for each individual.

We start by generating causal RVs. As RVs do not show high LD, we can model the population by generating each RV independently. We adapt Pritchard's argument that the frequency distribution of rare, deleterious, RVs must follow Wright's model under purifying selection [29]. Therefore, the allele frequencies 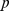 are sampled according to:
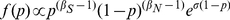
(7)where,

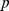
, allelic frequency

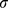
, selection coefficient

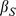
, rate of mutation from normal allele to causal

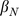
, rate of repair from causal allele to normal

We choose 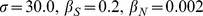 [29]. Note that we do not control the number of causal RVs, 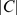, directly, in our simulation. Recall that
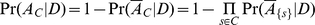
Further,
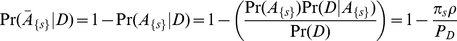
Therefore, setting a value for R limits the size of the causal RV.
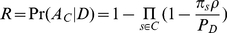
(8)Further, the sampling procedure occasionally generates SNPs with a high individual PAR. These variants would show up as being significant even with a single marker analysis. Therefore, these are discarded. The procedure SimulateRV describes the method to generate causal RVs. To generate neutral RVs, we use Fu's model of allele distributions [30] on a coalescent, which suggests that the number of mutations that affect 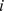 individuals in a population with mutation rate 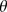 is given by 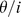. For the purposes of our simulation we use 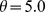.

**procedure** SimulateRV()

 Set 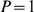

 Set 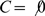

 Repeat

  Sample 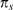 of low PAR 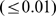 from Wright's distribution

  Generate a SNP 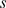 with MAF 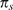

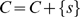

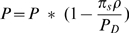

 while 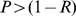

Generating Case-Control genotypes for RV simulation. Note that there is no explicit control of the number of causal RVs, but the choice of parameters helps to bound the number.

### Simulating genotypes

For both cases and controls, each RV is sampled independently. For non-causal variants 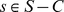, the probability of picking a minor allele is 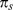, for both case and control individuals. To sample alleles from causal SNPs, recall that under the union model, 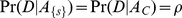 for all 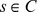. Therefore, the minor allele frequencies are given by
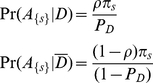
We assume HW equilibrium to sample genotypes for case and control individuals.

### Simulating populations with bottleneck and recent expansion (BRE)

Recently, Kryukov and colleagues [31] described a demographic model that explicitly models European ancestry. The population is assumed to be relatively stable for a long period, but is followed by a bottleneck, and rapid expansion after the bottleneck (about 7500–9000 years ago, with 20–25 years per generation). They validate their model by comparing observed versus predicted allelic frequencies. To this model, they add ‘causal’ (mostly deleterious) mutations using a distribution of selection coefficients from a gamma distribution. The causal alleles are associated with a change in a quantitative trait (QT). The QT values are normally distributed. Individuals carrying any causal mutation have QT values drawn from a Normal distribution with a shifted mean. For Rare variant analysis, individuals are chosen from the lower (Control) and upper (Case) tails of the QT distribution.

For our study, the authors provided us with individual genotypes simulated according to their demographic model, with causal mutations contributing to the following shifts: 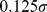 (Low), 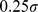 (Medium), 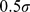 (High). The highest and lowest 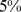, and 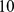
% of the QT distributions were used for the Case and Control populations. For the 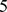
% population (500 controls, 500 cases), the locus PAR varied as 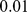
–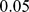. For the 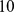
% populations, the number of individuals is larger (1000 controls, 1000 cases), but the PAR values decrease to 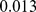 (Low), 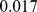 (Medium), and 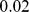 (High).

### Reimplementing alternative strategies

For the purposes of comparison, we reimplemented the collapsing statistic proposed by Li and Leal [15] as well as the weighted-sum statistic used by Madsen and Browning [16]. Both publications discuss the separation of variants into groups based upon function (i.e. non-synonymous coding SNPs) or other property. However, because we are performing our studies on model free, unannotated data, we do not perform any such grouping.

As a result, the CMC approach proposed by Li and Leal [15] is equivalent to collapsing all variants in the locus and calculating the association. Li and Leal show that the assignment of variants to functional groups, separately collapsing these groups, and finally performing a multivariate analysis improves power to detect causal loci. However, separation of variants into groups is inexact and the authors show that errors in group assignment can confound tests for significance. Additionally, performing this separation on a genome wide scale may be intractable.

The weighted-sum statistic proposed by Madsen and Browning [16] is used to detect association between a pre-defined group and a disease state. To compare fairly we defined the group of mutations as all mutations at a locus. We reimplemented the weighting approach based upon allele frequency as well as the sum and ranking approach to determine a score. Finally, we implemented a single-marker test as a bi-allelic 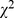-statistic with 1df. The tests were used to score windows over a wide range of simulation parameters to better understand how RareCover performed in comparison to the collapsing, and weighted strategies. For each strategy, a 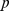-value of significance was established by doing 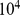 randomized trials using permuted case and control data. All three methods were run on the same sets of permuted data, and the 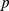-values were used to compare. Code for all methods is available upon request from the authors.

### MSMB gene resequencing

Recently, Yeager and colleagues [32] resequenced a 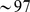
Kbp region including the micro-seminoprotein-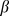 (MSMB) gene (chr10:
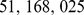
–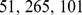) for 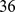 prostate cancer cases, 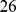 controls, plus another 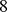 CEPH individuals. While the number of individuals is too small to derive rare variants, we used the prediced genotypes supplied by the authors for RV analysis. For this analysis, we used 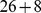 individuals together as controls, and all 284 variants with MAF 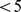
% were used as input to RareCover.

### CRESCENDO data

In a recently submitted study 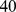 LR-PCR amplicons (Harismendy et al., unpublished) were used to re-sequence 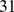
Kbp from the FAAH locus (NCBI36 chr1:46621328–46653043) and 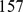
Kbp from the MGLL locus (NCBI36 chr3:128880456–129037011). A total of 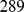 individuals were selected for sequencing from two tails of the BMI distribution of the CRESCENDO cohort (http://clinicaltrials.gov/ct/show/NCT00263042). 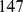 individuals had BMI lower than 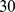 kg/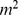 and 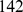 individuals a BMI greater than 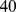 kg/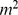. DNA sequencing libraries were prepared, and sequenced as previously described in Harismendy, 2009 [33] with the following modifications: sequencing libraries were indexed by 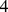
nt barcode located downstream of the adapter [34] and between one and six libraries were loaded per lane of the Illumina GAII instrument. The reads obtained from several lanes were merged, aligned and the variant called using MAQ mapmerge, map and cnsview+SNPfilter options respectively [35]. All samples had an average coverage greater than 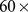. Allowing for a minimum coverage of 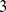 reads and a minimum base quality (Phred 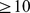), a raw set of 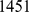 single nucleotides variants (SNVs) were identified in the population. The SNVs were filtered for Hardy Weinberg Equilibrium in the controls (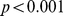) and genotyping rate 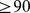
% of the samples to obtain a final set of 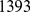 SNVs (220 FAAH, 1173 MGLL). Of these, 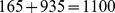 SNVs had MAF 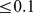, and were selected for RV analysis. The list and location of the RVs identified by RareCover as supporting the association is available in Supplemental Table S1.

## Results

### Simulations (CP)

We simulated cases and controls for a collection of sample sizes, ranging from 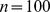 to over 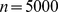 individuals with equal numbers of cases and controls. The MAF for rare variants ranged from 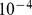 to 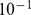. Throughout, we assume the disease prevalence in the population to be 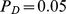. The PAR for the locus was set to 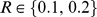. The penetrance, 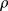 was varied in the interval 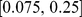, corresponding to OR values of 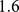
–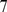. The dependence on parameters is somewhat non-trivial. To see this, note that 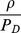 is a lower bound on relative risk. Reducing 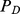 would increase the relative risk, only making association easier. In other words if the disease incidence is low, and a causal variant is low frequency, then the presence of the causal variant is a strong indicator of disease status.

**Figure 2:**
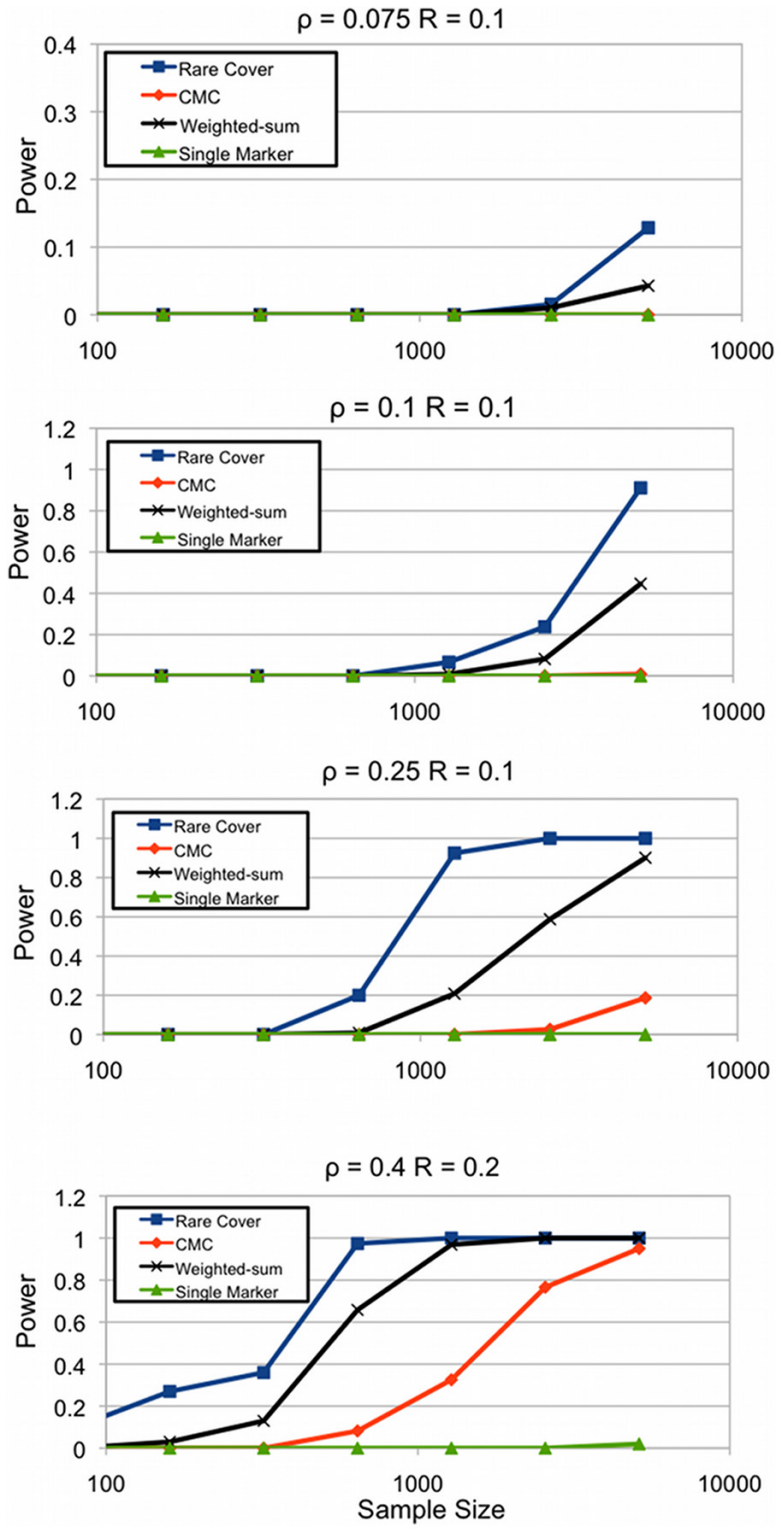
Power of RV analyses, tested over different values of penetrance 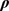, PAR 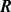, and 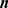 individuals (cases+controls). For each choice of parameters, 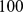 test cases were simulated. Each test-case was analyzed using 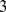 methods, and the 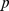-value computed using 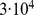 permutations of cases and controls. The score is considered significant only if it is higher than all permuted values. The power of the test is the fraction of test-cases that had a significant score. RareCover dominates the other methods implying greater power over all choice of parameters. For all methods, power increases with an increase in 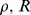, or sample size.

The results of the simulation are shown in Figure 2. For each choice of parameters (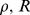, and 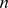), 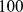 case-control studies were sampled as described in Methods. The data-set was tested using RareCover, collapsing, weighted-sum heuristics, as well as single-marker tests.

To enable a fair comparison, the 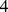 methods were applied to 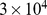 randomizations of the same data-set, obtained by permuting cases and controls. The 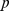-value is similar to a False Discovery Rate calculation. The span of a typical human gene is about 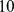
Kbp, and will contain about 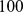 rare-variants, implying fewer than 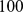 distinct windows per gene. If we assume 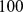 candidate genes for a phenotype, we would have 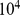 candidate windows. A 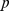-value, or FDR of 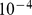 could therefore be considered significant at the genome-wide level. A test score was considered significant if it was higher than each of the 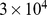 permutations, giving the 95% confidence interval of the p-value as 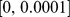. The power of a test for a specific choice of parameters is the fraction of (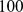) tests that had a significant score. Consider the sample point in Figure 2, with 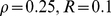, and a sample of 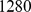 individuals. The power of RareCover is over 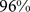, which can be contrasted with the low power of the weighted-sum [16], and collapsing heuristics. For any choice of parameters, RareCover shows better performance than the other methods.

Our phenotypic model differs somewhat from the one proposed by Madsen and Browning. In their model, the PAR for each causal variant is assumed to be equal, and is equal to the groupwise PAR divided by the number of causal variants. We also applied the tests to this model, using 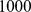 cases, and 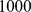 controls, and groupwise PAR values at 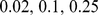. The power of the MB test at these values was computed to be 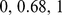 respectively, while the power of RareCover on the data sets is 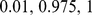 (Supplemental Figure S1).

**Figure 3:**
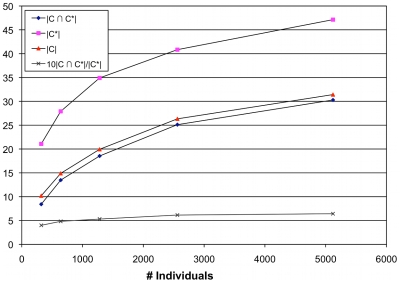
Comparisons between causal RVs, and RVs recovered by RareCover. The 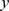-axis describes the raw number of causal RVs (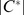), RVs recovered (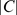), their intersection, and the fraction recovered (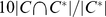, scaled for exposition). Close to 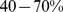 of the causal RVs are recovered over a wide range of sample populations.

An advantage of the RareCover approach is that it does not depend upon MAF, or the density of RVs in a region. This is partly because it combines the effects of multiple associating RVs that associate, and discards the RVs that do not associate. By contrast, other methods combine all RVs, albeit with different weights. While RareCover does not recover all of the causal RVs, it always recovered a significant subset of the causal RVs in our simulations. See Figure 3, which summarizes the results for 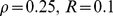. Let 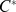 correspond to the simulated, causal RVs, while 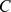 corresponds to the set returned by RareCover. Thus, 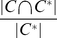 corresponds to the fraction of causal RVs recovered. With modest sample size, more than 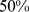 of the RVs are recovered, and help provide a direct interpretation of the genetic association. A somewhat unexpected aspect is that the number of causal RVs, 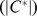, (and also, 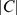) increases with an increasing sample size. For larger samples, we can recover a larger number of the low frequency variants, and the causal set has a larger mix of low frequency RVs. As we only consider RVs with MAF 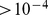, the number saturates by 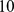
K individuals.

### Simulated BRE populations

The 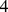 methods were also applied to the data sets provided by Kryukov et al., as explained in Methods. The cases and controls are chosen from the extremes of a population of 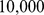 phenotyped individuals to reflect current population cohorts. As the locus PARs are very small (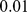
–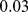), we work with a nominal 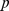-value cut-off of 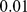. As before, power is defined as the fraction of 1000 simulations on which the test met the 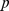-value cut-off. In addition to the 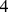 methods, we also plot the power of the true causal mutations to illustrate their small effect.

**Figure 4:**
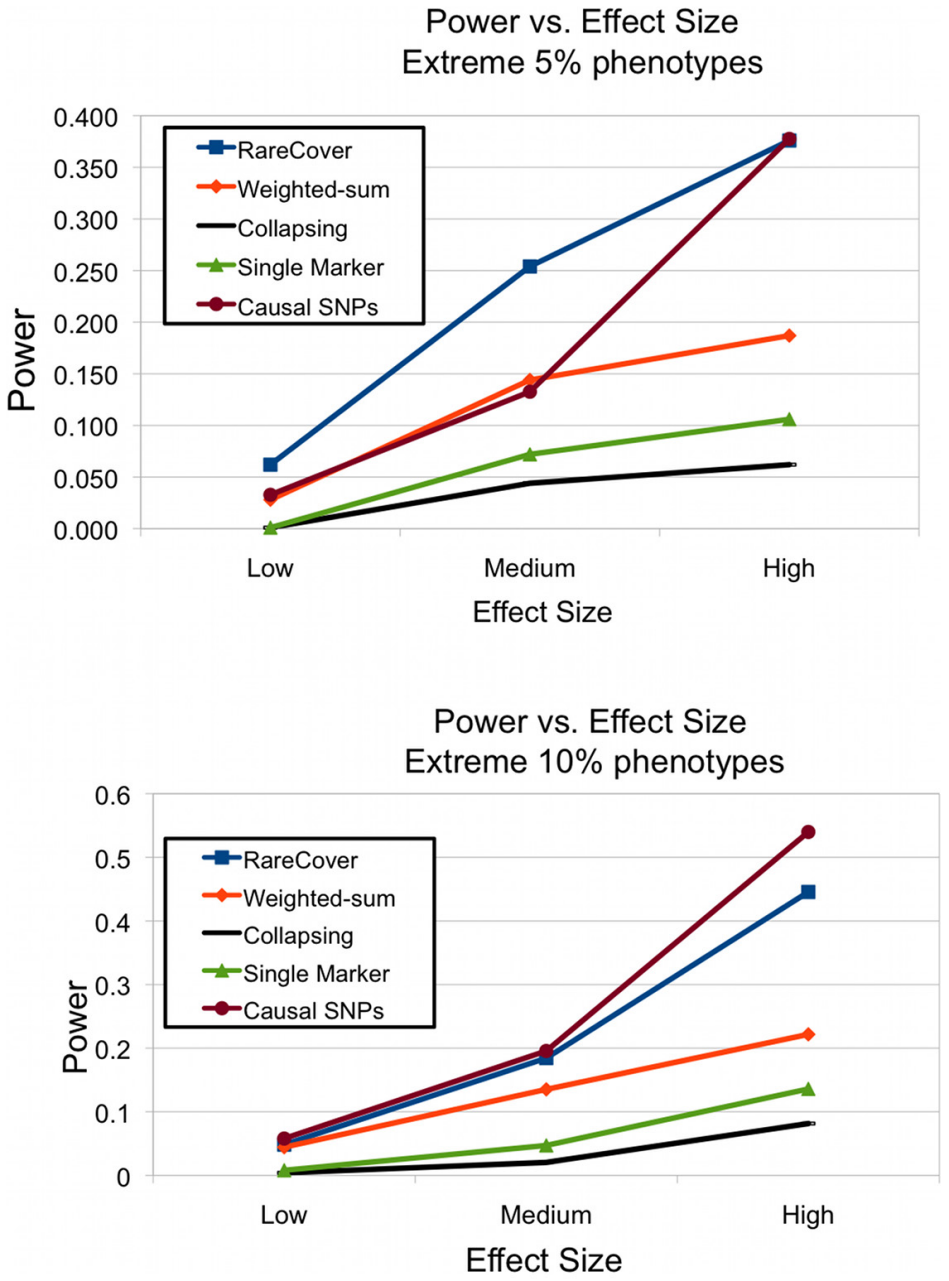
Power calculations on populations with bottleneck, and recent expansion. Simulated population data with quantitative trait (QT) values was provided by Kryukov et al. The QT values are normally distributed. Individuals carrying any causal mutation have QT values drawn from a Normal distribution with a shifted mean. The shift is characterized as Low (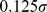), Medium (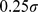), and High (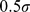). As the locus PAR values are low, power is computed as the fraction of 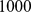 simulations that showed significance at 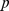-value 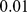. Individuals were chosen from the lower (Control) and upper (Case) tails of the QT distribution. The power of all methods is compared using the 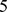
% extremes (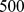 cases, 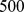 controls), and the 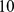
% (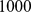 cases, and 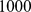 controls). RareCover is shown to have the highest power, comparable to the power of the causal mutations.

Figure 4 shows the results upon choosing the 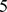
%, and 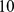
% extremes for different levels of phenotypic association. RareCover outperforms other methods over the different tests, and is comparable to the results of selecting the true causal mutations. As suggested previously, increasing PAR values, and population sizes, increase the power of RareCover, as with all methods. However, the power of RareCover does not appear to be affected by the specifics of the demographic simulation.

**Figure 5:**
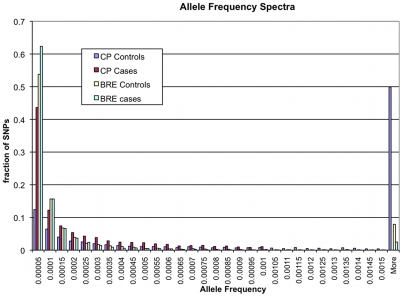
Allele frequency spectra in various demographic models. BRE refers to the simulation of population under bottleneck followed by recent expansion from Kryukov et al.; CP refers to the simulation under a constant population size. The allele frequencies in CP are biased toward rare variants in cases, while there is little bias in BRE. The performance of RareCover is robust to data sets with different allele frequency spectra.

The allele frequency spectrum of the CP and BRE models is shown in Figure 5. There are significant differences in allele frequency spectra in the two cases. In the CP case, there is a bias in the cases among alleles with lower frequency. High frequency causal variants represent an easier case, as they can be detected by single marker analysis. To eliminate these cases, we discarded high frequency causal variants from the simulations, which partly explains the bias in CP, relative to BRE. In the CP (respectively, BRE) models, the average number of variants per 5Kbp window was 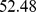 (respectively, 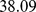), with 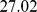 (respectively, 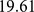) causal variants. The performance of RareCover is robust against different demographic models, and depends mainly upon locus PAR, and sample size.

### Running time

**Figure 6:**
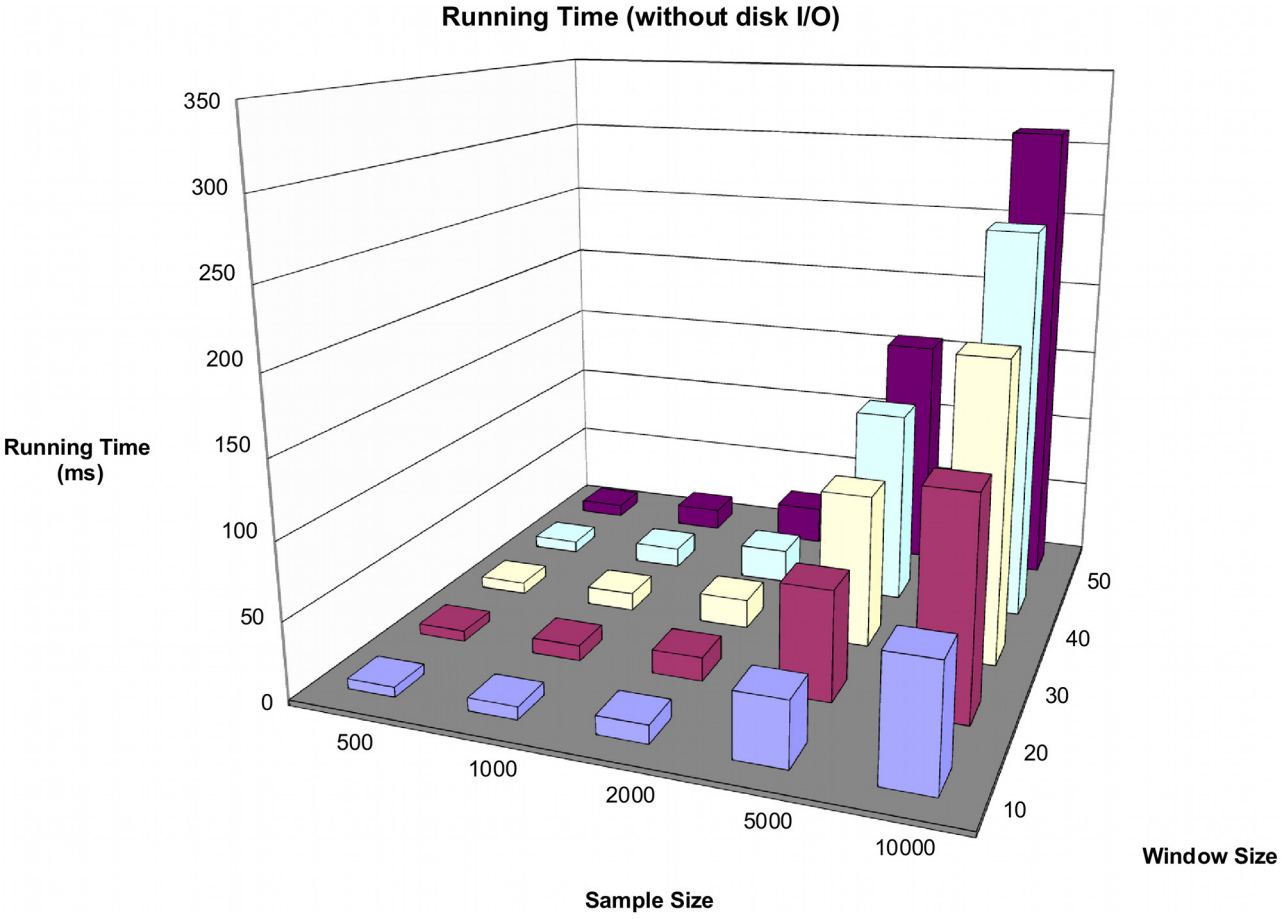
Running time of RareCover as a function of sample size, and number of SNPs. As RareCover is a greedy approach, the running time increases linearly with an increase in number of SNPs, and individuals. The running time shown here does not include the time for disk input and output of the data, which incurs a fixed additional cost of 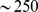
ms to each run. The total running time is about twice that of single marker tests.

The running time of RareCover increases linearly with the number of SNPs, and the number of individuals, as shown in Figure 6. For a population of 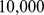 individuals, the running time time goes from 80ms to 311ms on a standard Linux desktop, as the number of SNPs in the window increases from 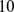 to 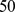. The times shown here do not include the cost of reading and writing the data, which incurs a fixed additional cost (about 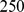
ms. See Supplemental Figure S2). The total running time is at most 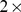 that of a single marker test.

On the FAAH data (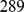 individuals), the running time for a window of 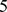
Kbp was computed to be 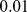 seconds. Consider a genome-wide scan with 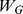 windows. To achieve genome-wide significance, we would need 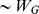 randomizations for each window, which could be computationally intensive.

However, we run RareCover in two passes, using the Xcorr statistic on the union-variant as a filter (Figure 1). The permutation test is only applied to the fraction 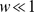 of the 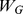 windows for which the Xcorr statistic exceeds a threshold (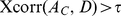).

Therefore the RareCover computation is executed on 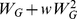 windows. As discussed in the methods, 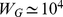. For 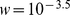 (corresponding to 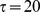 in Figure 1), the total time is
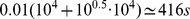
If the number of candidate windows is larger (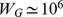), and a conservative filter is chosen (corresponding to 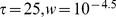), the running time increases to 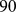 hrs., easily accomplished on a small cluster.

### Results on resequencing data

#### MSMB data

A RareCover analysis of the 284 variants did not identify anything significant. A 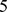
Kbp window starting at chr10:51253758 has a nominal 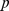-value of 0.06. The 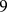 SNPs selected by RareCover cover 12 cases and 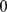 control individuals. If we apply RareCover after including common variants, the same region has a nominal 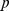-value of 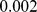 due to a common variant that occurs in 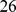 cases, and 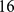 controls. This window lies not in MSMB but in a neighboring gene, NCOA4, also a candidate gene for prostate cancer risk [32]. A larger population sequencing will help resolve if this is a true association.

#### CRESCENDO cohort data

**Figure 7:**
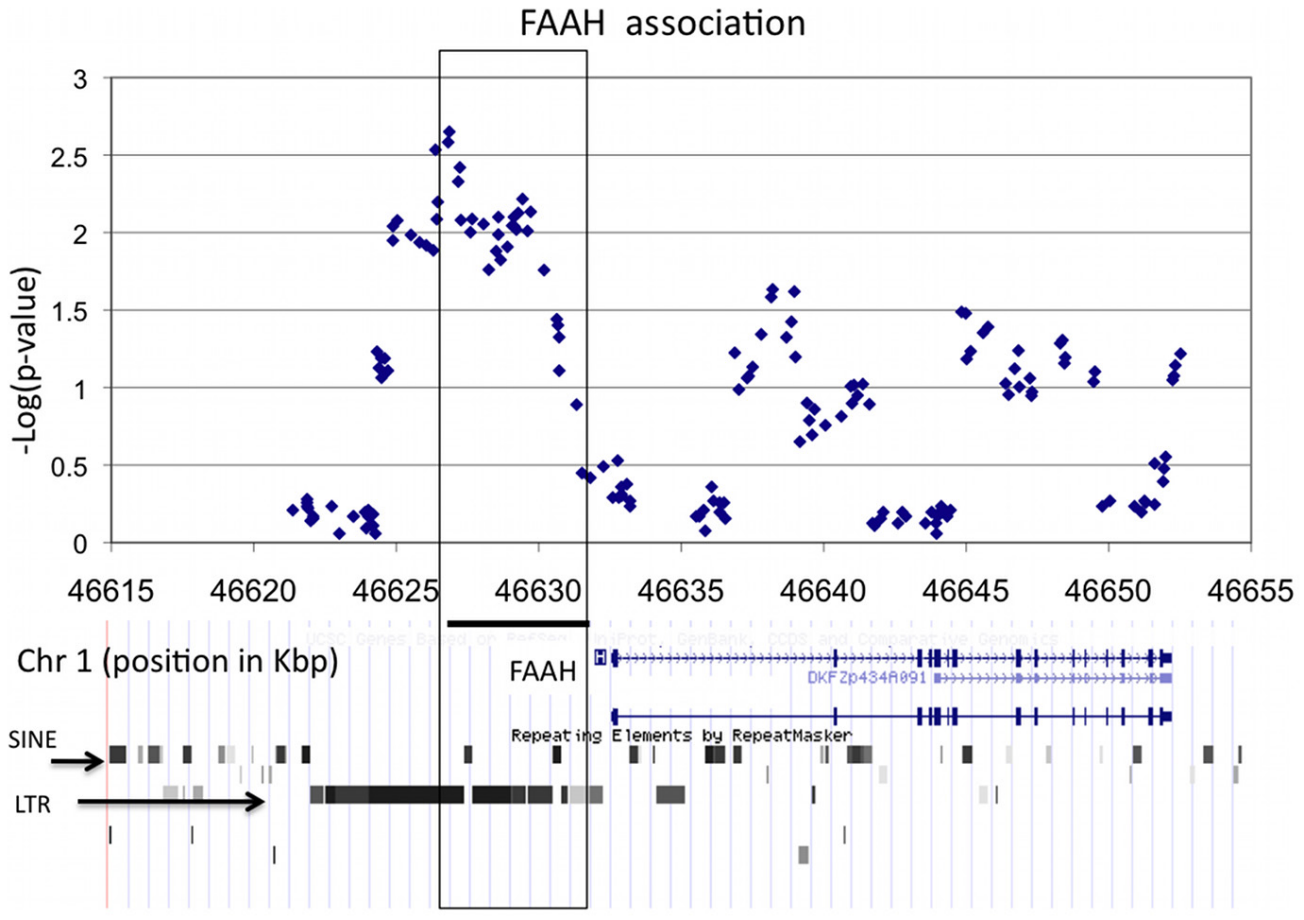
FAAH locus association. RareCover was used to analyze overlapping windows of 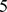
Kbp in the re-sequenced region around FAAH. A 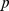-value was computed for each window using 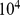 permutations of cases and controls. Each point corresponds to the 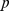-value of a single window starting at that location. The most significant window (described by the box) is 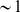
Kbp upstream of the FAAH transcription start site. The region is part of an LTR element, which are known to carry regulatory signals, and is enriched in transcription factor binding sites, suggesting a regulatory role for the rare variants.

As described earlier, the CRESCENDO cohort subjects selected for sequencing were individuals at the extremes of BMI distribution. We applied RareCover to overlapping windows of length 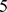
Kbp over the region (as described in Methods), to analyze the impact of RVs (For the purposes of comparison the performance of all methods can be seen in Supplemental Figure S4). The permutation based 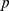-values for two genes are plotted in Figure 7. For both loci, we found a single region of approximately 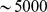
bp, that was enriched in strongly associating variants.

The FAAH enzyme (1p33) is known to hydrolyze anandamide (AEA), and other fatty acid amides. The region with the most significant association, located 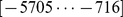 upstream of the FAAH transcription start site (TSS), contains 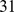 RVs. RareCover selected a subset of 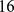 RVs, with a union-variant that appears in 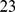 cases, and 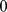 controls (nominal permutation 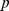-value 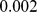). The locus specific 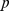-value for the window is 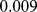. Analyzing the locus for functional significance, the locus falls within a retroviral Long-Terminal-Repeat (LTR). Insertion of retroviral elements, followed by adaptation of the viral regulatory elements is a well known mechanism for gene regulation. A recent analysis of the FAAH core promoter (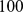
bp upstream) in human T-cells identified a C/EBP site which (through a STAT3 tethering) mediated the leptin regulation of FAAH expression [36]. Surprisingly, the leptin mediated regulation of FAAH was observed in immune cells, but not a model of neuronal cells [37]. Our results suggest that an alternative regulatory region 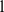
Kbp upstream of the TSS is disrupted by RVs in obese individuals. A scan for transcription factor binding sites reveals many relevant transcription factor binding sites, including one for C/EBP (data not shown).

The enzyme monoacylglycerol lipase (MGLL), encoded by the MGLL gene located on chromosome 3q21.3, is a presynaptic enzyme that hydrolyzes 2-arachidonoylglycerol (2-AG), the most abundant endocannabinoid found in the brain. The RareCover scan on 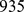 RVs identified a single window enriched with associated RVs. The window lies immediately upstream of the gene, suggesting that the causal RVs have a regulatory function. At the most significant locus (chr3:
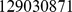
–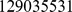, upstream of MGLL TSS), 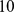 of 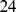 RVs were selected, with the union RV present in 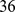 cases, and 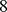 controls. While the nominal 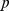-value is 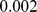, the locus adjusted 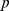-value is at the margin of significance, at 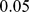. The locus contains a known LINE element and a promoter for RNA polymerase II. Mutations in this promoter could easily interfere with binding affinity for RNA Polymerase II and affect subsequent transcription/translation.

In our analysis, we only considered RVs (MAF 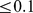). Harismendy et al. have reported on the connection between common variant, and RV associations in a recently submitted study. Their results suggest that common variants do not identify significant associations in FAAH, but identify 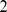 regions with significantly associated SNPs at the MGLL locus. LD between the significant common variants and the MGLL union-variant is low, with the highest 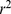 value of 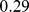. This suggests that the rare and common variations might have independent, additive effects on the phenotype.

## Discussion

We described a novel method for Rare variant analysis with greatly improved power of detecting associations, relative to other published methods. RareCover utilizes the specific properties of RVs as compared to common variants, and applies a greedy approach to picking a subset of RVs, that best associate with the phenotype. It is a natural extension of previous methods, which either collapsed all RVs at a locus, or collapsed them after weighting different SNPs differently. Our algorithm is similar in orientation to the greedy solutions for the combinatorial problems of identifying set-cover and test-cover (See for example, Lovasz [38]). However, it is specifically designed for case-control analysis. The power of the method is extensively analyzed against different values of locus PAR, penetrance, and sample size. RareCover easily outperforms other methods which group, and collapse SNPs. The weighting approaches are reasonable, given that most causal RVs have functional significance, and likely to have moderately high penetrance, which one would not expect in a non-causal RVs. However, a large number of non-causal RVs, even with small weights, can dilute the association of the causal RVs. Also, it is difficult to identify different groupings of RVs, and to set appropriate weights for different groups.

Our application of the method on the CRESCENDO individuals, generates plausible hypotheses on the role of FAAH and MGLL in of obesity. The genetic association of FAAH with obesity is interesting because many previous studies with common variants have failed in identifying significant associations. We investigated the hypothesis that alleleic heterogeneity due to multiple RVs, influences the obesity phenotype. Second, the low LD between RVs and causal variants implies that if an RV is significantly associated, it is likely to have functional significance. Our simulations confirmed that RVs identified by RareCover were enriched in the causal RVs. In analyzing FAAH and MGLL, we identified exactly one small, functionally significant region, at each locus with significant association. This suggests that multiple rare variations help influence the regulation of the two genes. Recently, Sipe and colleagues collected metabolite expression levels on 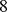 metabolites from 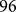 severely obese subjects and 48 normal weight subjects [39]. Comparing against our FAAH data, we find that the levels of AEA (anandamide) are highest in obese individuals that carry an RV identified by RareCover, and lowest in individuals that are non-obese, and do not carry the causal RV. As FAAH helps metabolize AEA (anandamide), this result is consistent with the hypothesis of the RVs disrupting FAAH expression. The data on all metabolite expression will be published elsewhere (Harismendy et al., unpublished).

Nevertheless, our study also raises many methodological questions. Our approach is greedy, in that it selects the most discriminating RV at each step. Theoretically, it is possible that a collection of RVs, that are individually less discriminating, are jointly more strongly correlated. In that case, RareCover will not identify them. We implemented an approach based on simulated annealing to find an optimal subset of SNPs. However, in our simulations with the union model, the greedy method worked as well as the more complex optimization, and was significantly faster. Recall that in the simple Union model, the penetrance does not change upon inclusion of additional SNPs, but the PAR increases. Other, more complex models are possible. For example, we could have a threshold model, in which the penetrance increases with a minimum number of rare alleles. Or, we could have additive models, where the penetrance increases as a function of the number of rare alleles. As more re-sequencing data becomes available, these will be the focus of additional investigation. A second issue is that our definition of a locus is set arbitrarily as a window of fixed length, much like in other methods. However, empirical tests with a small range of window-sizes did not significantly change the results. It is possible that a dynamic assignment of the size of the locus could increase power, but at the cost of additional computations.

In this study, we analyze only the rare variants. While the RareCover algorithm can work unchanged with rare and common variants, a correct test for power of such an approach would require a biological model that combines the effect of RV and common variants. It is hard to speculate on such models in the absence of empirical data. However, preliminary results on comparing common and rare variants at the MGLL locus suggest an independent, additive effect.

GWA studies have shown that identifying the genetic basis of disease depends upon many factors. For this reason, algorithms have been devised to deal with population substructure issues, epistatic interactions between loci, as well as rare variant analysis. Our results indicate that RV analysis is useful in many contexts, and novel methods may have to be developed to include the effect of RVs in all of the above.

## Supporting Information

Figure S1 Madsen and Browning models. RareCover performance on the phenotypic models proposed by Madsen and Browning. In this model, the PAR for each causal variant is assumed to be equal, and is equal to the groupwise PAR divided by the number of causal variants. The power of RareCover and other methods is applied on populations with 1000 cases, and 1000 controls, and groupwise PAR values at 0.02, 0.1, and 0.25.
(0.46 MB TIF)

Figure S2 RareCover running time including I/O. Running time of RareCover as a function of number of individuals, and number of SNPs, including time for input and output of data. The time for input and output dominates when the number of individuals is less than 2000. Otherwise, the time increases linearly with an increase in number of SNPs, and number of individuals.
(1.51 MB TIF)

Figure S3 RareCover on MGLL. Performance of RareCover on MGLL. The most significant window (described by the box) appears upstream of the MGLL gene, near the promoter region.
(0.55 MB TIF)

Figure S4 Method comparison. Performance of RareCover the weighted-sum statistic, and collapsing on the FAAH and MGLL. Some peaks are replicated in only a subset of methods. RareCover is the only method that identifies a significant hit in a region in MGLL containing common variants associated with the disease phenotype. Common variants were excluded from this analysis.
(1.62 MB TIF)

Table S1 Detailed SNP information for the windows highlighted in Figure 3 and Figure S3. The columns indicate the SNP id, position, and relative risk of each SNP within the selected 5000 bp windows. Case Matches refers to the number of case samples that carry the SNP, and similarly for Control Matches. The worksheets containing raw data give the genotype at each SNP (0,1, or 2) within each 5000 bp window as well as disease status. Individual ids have been removed.
(0.15 MB XLS)
