# Supplementary figures and images for: A Covering Method for Detecting Genetic Associations between Rare Variants and Common Phenotypes
Source: PLoS Comput Biol. 2010 Oct 14;6(10):e1000954. doi: 10.1371/journal.pcbi.1000954 (PMC2954823; doi:10.1371/journal.pcbi.1000954)

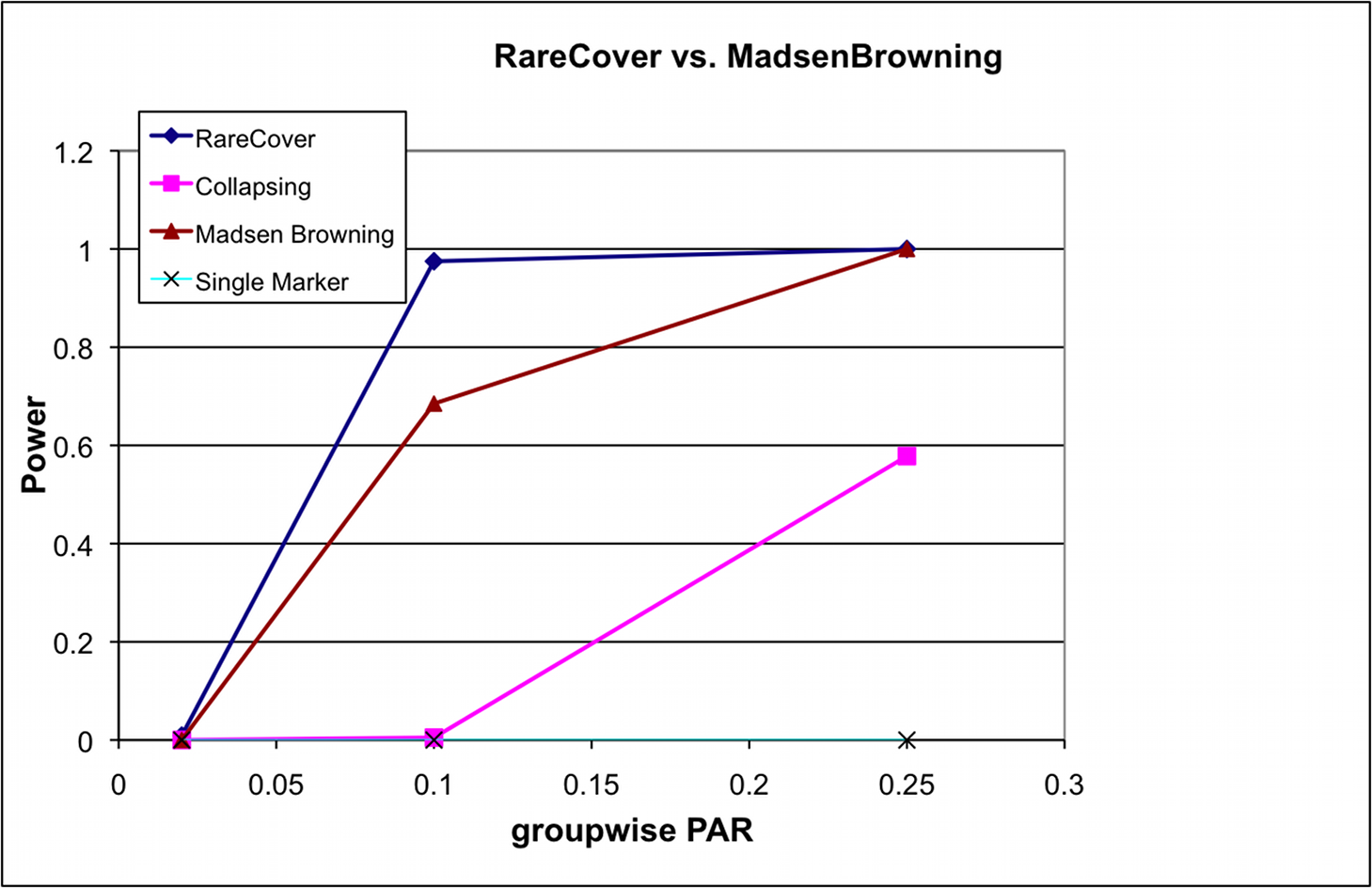

Supplement: Figure S1 — Madsen and Browning models. RareCover performance on the phenotypic models proposed by Madsen and Browning. In this model, the PAR for each causal variant is assumed to be equal, and is equal to the groupwise PAR divided by the number of causal variants. The power of RareCover and other methods is applied on populations with 1000 cases, and 1000 controls, and groupwise PAR values at 0.02, 0.1, and 0.25. (0.46 MB TIF) [file pcbi.1000954.s001.tif]

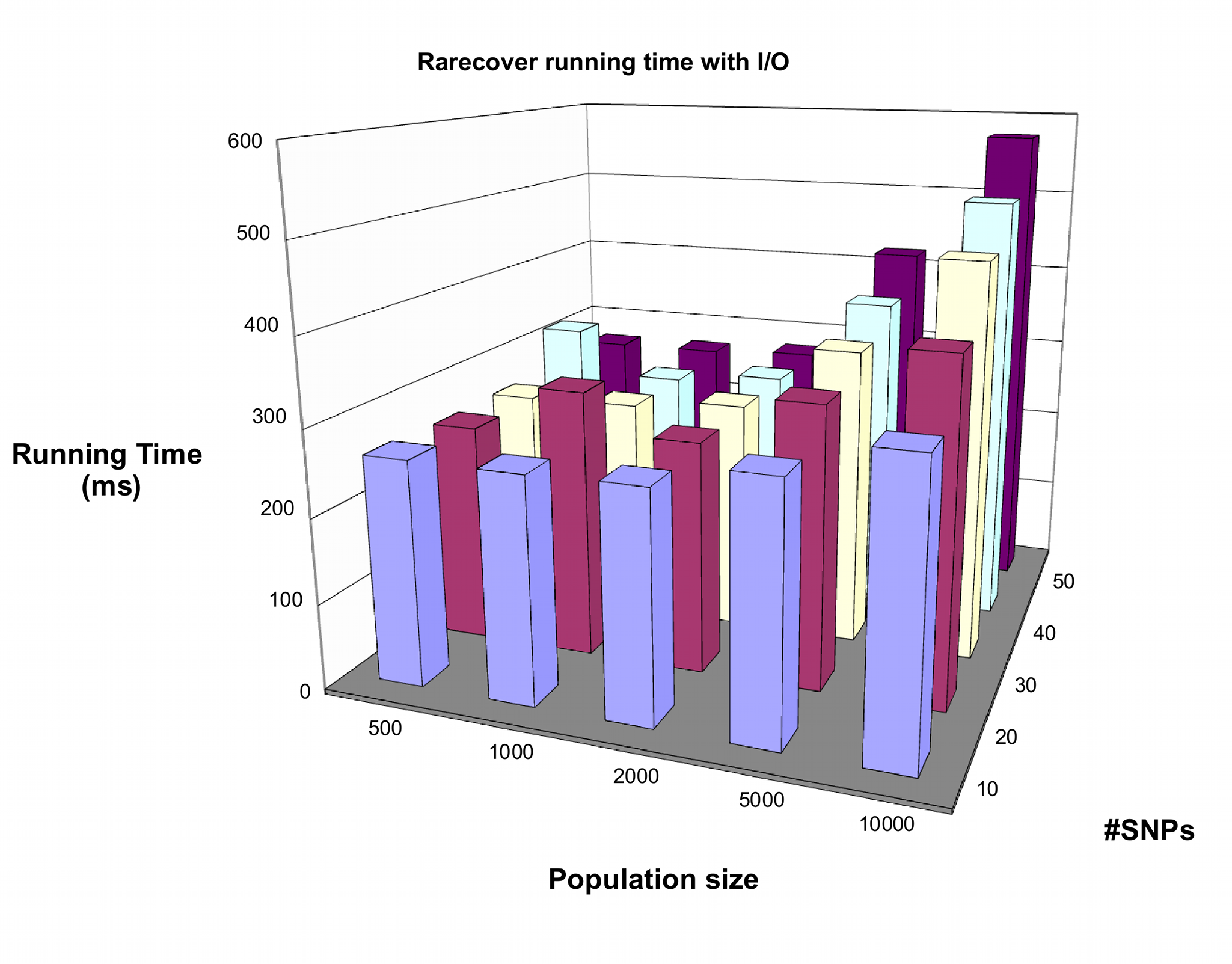

Supplement: Figure S2 — RareCover running time including I/O. Running time of RareCover as a function of number of individuals, and number of SNPs, including time for input and output of data. The time for input and output dominates when the number of individuals is less than 2000. Otherwise, the time increases linearly with an increase in number of SNPs, and number of individuals. (1.51 MB TIF) [file pcbi.1000954.s002.tif]

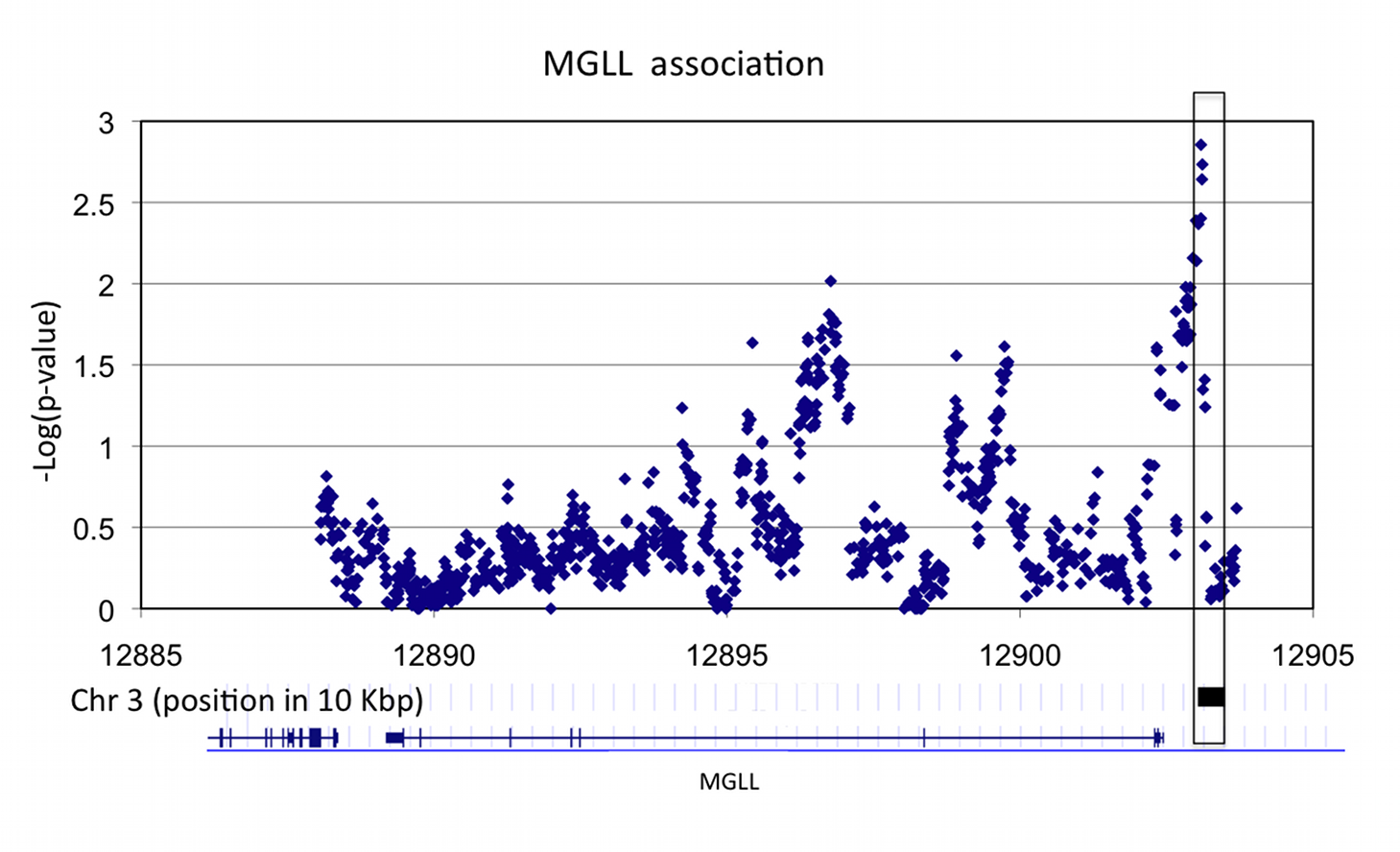

Supplement: Figure S3 — RareCover on MGLL. Performance of RareCover on MGLL. The most significant window (described by the box) appears upstream of the MGLL gene, near the promoter region. (0.55 MB TIF) [file pcbi.1000954.s003.tif]

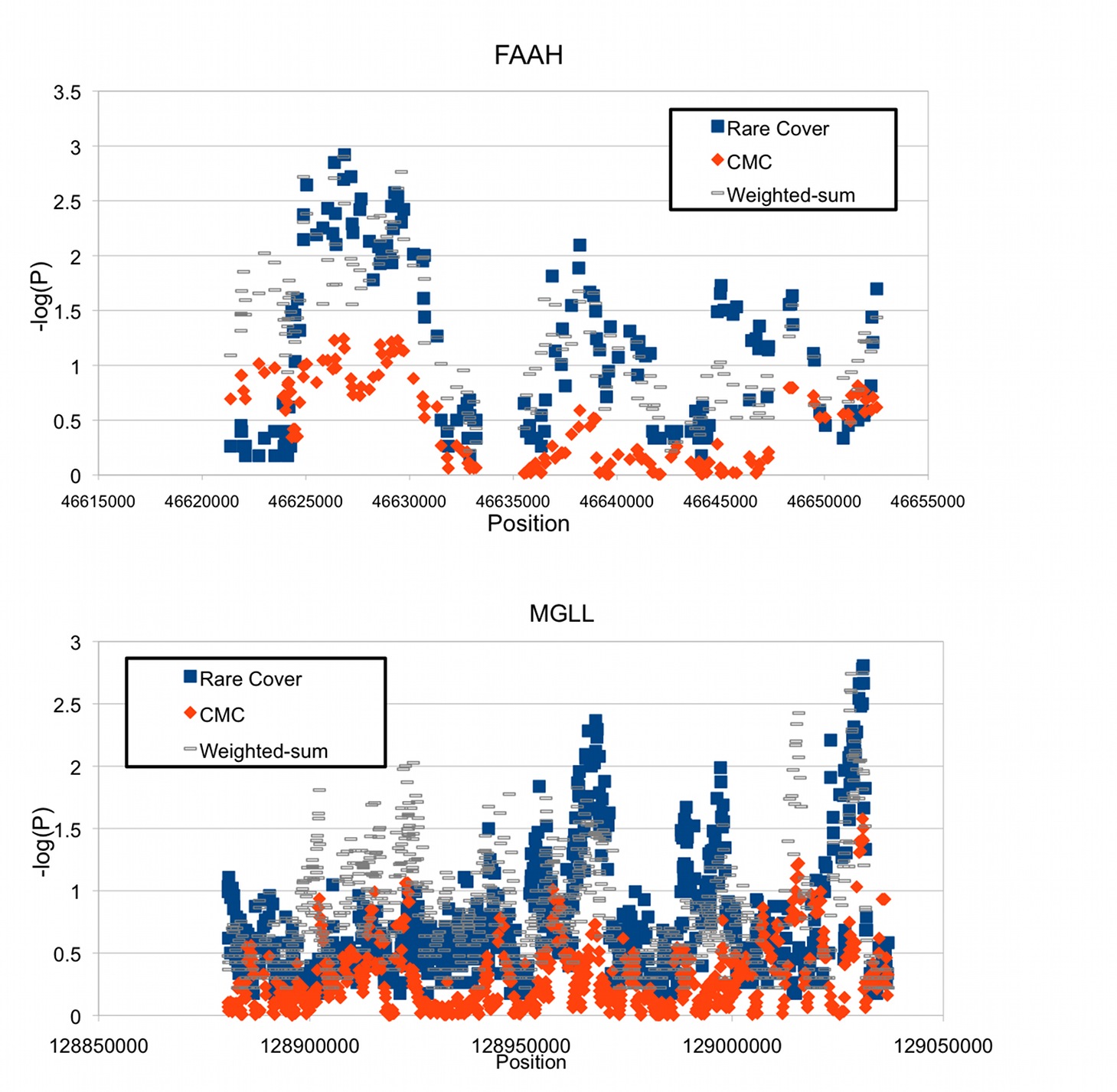

Supplement: Figure S4 — Method comparison. Performance of RareCover the weighted-sum statistic, and collapsing on the FAAH and MGLL. Some peaks are replicated in only a subset of methods. RareCover is the only method that identifies a significant hit in a region in MGLL containing common variants associated with the disease phenotype. Common variants were excluded from this analysis. (1.62 MB TIF) [file pcbi.1000954.s004.tif]
